# Adolescent idiopathic scoliosis with rapid progression – a case report

**DOI:** 10.1186/1748-7161-7-S1-P17

**Published:** 2012-01-27

**Authors:** M Tyrakowski, T Kotwicki, J Czubak

**Affiliations:** 1 Depatment of Orthopaedics, Pediatric Orthopaedics and Traumatology of Postgraduate Medical Education Centre in Warsaw, Otwock, Poland; 2 Spine Disorders Unit, Department of Pediatric Orthopedics and Traumatology, University of Medical Sciences, Poznan, Poland

## Background

Adolescent idiopathic scoliosis in immature patient is likely to progress when exceeds 30 degrees of Cobb, however the progression rate is usually moderate, amounting to several degrees per year [1,2].

The aim of the study is to present a patient with untypically rapid progression.

## Case report

A 15-year-old girl having right thoracic scoliosis with documented progression of 100 Cobb degrees within 2 years is presented.

Medical history: Until the age of thirteen the girl developed normally, then the parents noted trunk asymmetry. The girl was radiographed and right thoracic scoliosis was diagnosed. Cobb angle was 20 degrees (Th7-L2), Risser sign 1. No signs of congenital curvature. The girl was advised to wear a brace and perform exercises but did not do.

First admitted to our department at the age of 15. Menarche has not appeared. On examination severe trunk imbalance, right rib hump 30 degrees of Bunnell. The curve was stiff, left rib arch touching the iliac crest. There were 4 café-au-lait spots, but diagnose of neurofibromatosis was ruled out with genetic exam. X-ray revealed right thoracic scoliosis 125 degrees of Cobb (Th7-L2), Risser sign 3. Supine bending X-ray revealed correction to 100 degrees (20%). Vital capacity was 40%. MRI exam did not show central nervous system disturbances.

The patient was qualified to staged operative treatment of scoliosis combined with regular respiratory exercises and had already the first surgery resulting in correction to 67 degrees.

## Conclusions

Medical history, clinical examination, radiography and MRI did not reveal any data to predict unusual course of scoliosis.
